# Corrigendum for The clinicopathological and microRNA expression signature associated with lymphovascular invasion in squamous cell carcinoma: a basic descriptive study

**DOI:** 10.1002/hsr2.989

**Published:** 2022-12-15

In the article Robison et al.^1^, the first author's name was spelled incorrectly. The correct author's name is Shayene Robinson.

Robison, S, Ngwenya, S, Molaudzi, M, Molepo, J, Adeola, H, Magangane, P. The clinicopathological and microRNA expression signature associated with lymphovascular invasion in squamous cell carcinoma: a basic descriptive study. *Health Sci Rep*. 2022;5:e958. doi:10.1002/hsr2.958

We apologize for this error.

